# A high-resolution large-scale dataset of pathological and normal white blood cells

**DOI:** 10.1038/s41597-023-02378-7

**Published:** 2023-07-19

**Authors:** Alexandra Bodzas, Pavel Kodytek, Jan Zidek

**Affiliations:** Department of Cybernetics and Biomedical Engineering, Faculty of Electrical Engineering and Computer Science, VSB-Technical University of Ostrava, Ostrava, Czech Republic

**Keywords:** Biomedical engineering, Cell biology, Cells

## Abstract

Microscopic examination plays a significant role in the initial screening for a variety of hematological, as well as non-hematological, diagnoses. Microscopic blood smear examination that is considered a key diagnostic technique, is in recent clinical practice still performed manually, which is not only time consuming, but can lead to human errors. Although automated and semi-automated systems have been developed in recent years, their high purchasing and maintenance costs make them unaffordable for many medical institutions. Even though much research has been conducted lately to explore more accurate and feasible solutions, most researchers had to deal with a lack of medical data. To address the lack of large-scale databases in this field, we created a high-resolution dataset containing a total of 16027 annotated white blood cells. Moreover, the dataset covers overall 9 types of white blood cells, including clinically significant pathological findings. Since we used high-quality acquisition equipment, the dataset provides one of the highest quality images of blood cells, achieving an approximate resolution of 42 pixels per 1 μm.

## Background & Summary

Bone marrow disorders usually manifest by qualitative, as well as quantitative alterations of blood elements. Blood cells can moreover serve as easily accessible indicators of diseases that affect diagnostically challenging organs, where biopsies are difficult or risky to obtain^1^. Therefore, analyzing the characteristics of blood cells at different stages of differentiation often has a decisive impact and plays a critical role in the diagnosis. Due to this fact, microscopic examination of peripheral blood smears remains, despite the technological advances, a frontline diagnostic technique that not only allows clinicians to assess the patients’ health but also enables them to monitor the effectiveness of the treatment^1^.

Although the traditional manual blood smear examination is considered the gold standard in hematology, the manual examination comes with numerous drawbacks, such as strong subjectivity or poor standardization of results. In addition, manual microscopic examination is a time-consuming process that requires a considerable amount of experience and can lead to incorrect interpretations or misdiagnosis due to human errors^2^. To overcome the above-mentioned limitations and minimize human intervention, automated and semi-automated systems have been developed in the last decades to provide much faster, more objective, and standardized analysis. Even though many laboratories have integrated these technologies into their diagnostic procedures, the high costs associated with purchasing and maintaining these systems make them unavailable for smaller and medium-sized clinical facilities and laboratories.

Extensive research has been recently conducted to explore the possibilities of a more accurate and feasible solutions. One of the greatest problems researchers face in this field is a limited availability of medical data and extensive high-quality datasets. In the worst case many authors verify their proposed solutions on a small local and publicly unavailable datasets^3–9^, which makes it impossible for other researchers to compare their findings with existing studies. Despite the availability of annotated blood cell datasets from several studies, or publicly available repositories, which typically consist of only a few hundred^10–13^ or rarely a few thousand images^14^, the majority of researchers in this field still depend on data augmentation techniques to expand their datasets. Although data augmentation is a powerful technique to increase the size of the datasets and enhance the generalization of machine learning models, it should be noted that expanding datasets by augmentation techniques carry the potential risk of not capturing all the possible variations and complexities of the real-world data. Some existing studies^15,16^ have even published images from automated cell analyzers, such as CellaVision. However, while these datasets may contain a higher number of samples, they may not sufficiently capture the authenticity and variability of manually stained blood smears. Another shortcoming of existing datasets may be the lack of pathological findings, i.e., immature white blood cells, which are often missing or occur at low frequencies within the datasets.

To address the deficiency of extensive databases and support the ongoing research, we created a high-resolution dataset containing a total of 16027 annotated normal and pathological white blood cell samples. The dataset contains overall 9 types of white blood cells, including particularly neutrophil segments and bands, eosinophiles, basophiles, lymphocytes, monocytes, nucleated red blood cells (NRBC), sometimes termed as normoblasts and leukemic cells of both, myeloid and lymphoid lineage. Additionally, the images in the provided dataset capture various lighting conditions and blood film artifacts that can arise from manual blood smear preparation and staining. Unlike in other conducted studies that publish datasets, the data acquisition in this experiment was performed using high-quality acquisition equipment, which resulted in an approximated image resolution of 42 pixels per 1 μm. This resolution is the highest achieved quality of blood cell images from manually stained blood films examined under the microscope that the authors of this study are aware of.

## Methods

During this study conducted between years 2020 and 2022, a total of 12986 microscopic blood smear images were collected from 36 peripheral blood smears with diagnosed leukemia and 45 smears without leukemic pathology. These samples were collected from 78 anonymized patients, who participated in the study. Among them, 18 patients were diagnosed with acute myeloid leukemia, 15 patients suffered from acute lymphoid leukemia, and 45 patients did not show any pathological findings or had a non-leukemic diagnosis. All blood smear samples were stained manually with May-Grünwald and Giemsa-Romanowski staining solution, which enable differentiation of white blood cells. The lineage of the blast cells was in all cases determined by flow cytometry. Since cell morphology does not carry relevant information about specific leukemia subtypes in recent clinical practice, the dataset does not include further details about patients’ diagnoses. All data were collected in accordance with the relevant guidelines and regulations. The ethical approval was obtained from the ethical committee of the Ostrava City Hospital, Czech Republic. Since all data come from archived blood smear samples from anonymized patients, the ethics committee ruled that no consent from participants was required in this study.

### Data acquisition

**Fig. 1:**
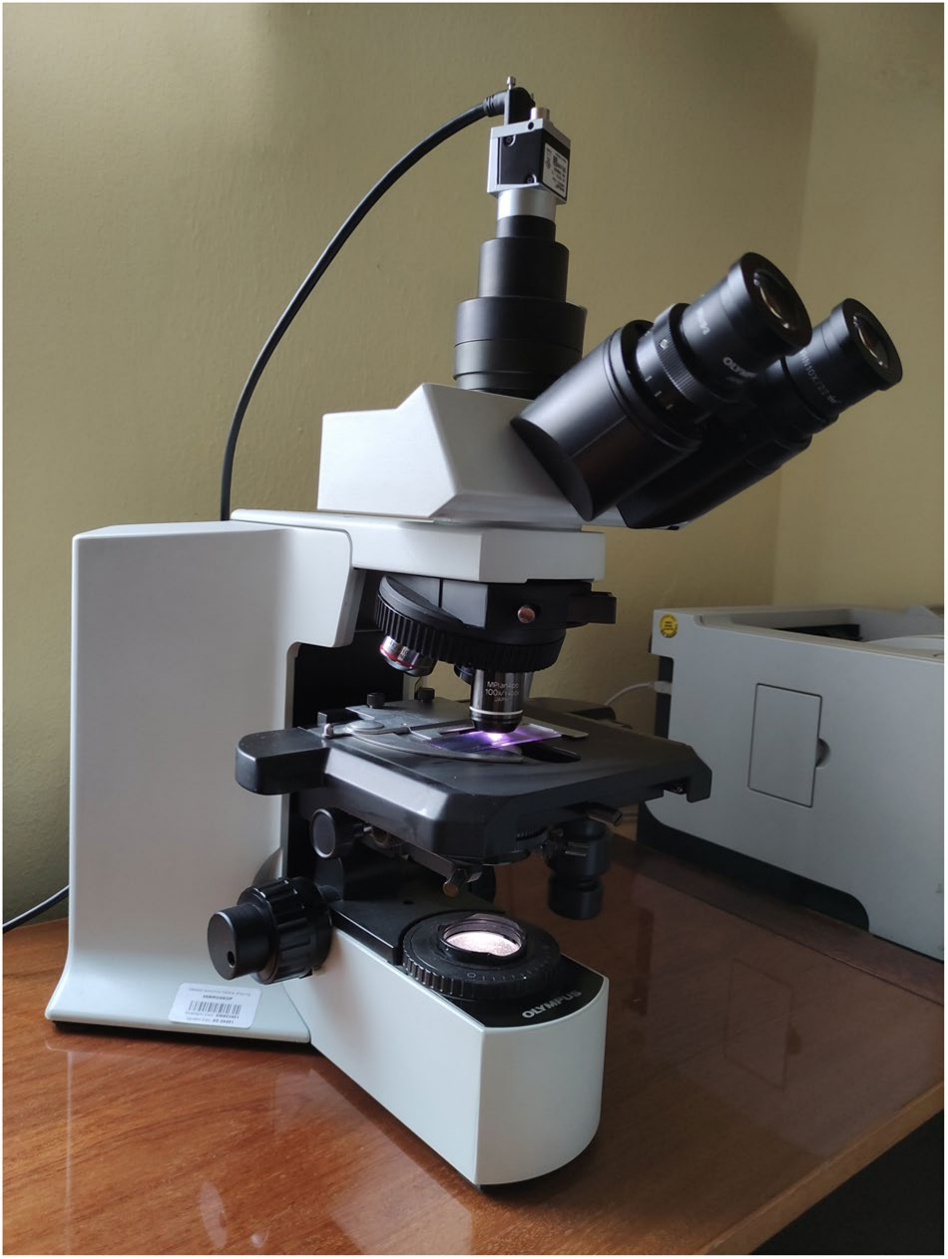
The acquisition hardware setup including the Olympus BX51 microscope and a high-resolution color camera Basler acA5472-17uc.

The data acquisition in this study was carried out by a simple hardware setup consisting of an Olympus BX51 microscope and a mounted high-resolution color camera Basler acA5472-17uc with a frame rate of up to 35 frames per second. This sampling frequency allowed us to achieve a relatively fast image response time during the manipulation with the examined sample, which speeded up the whole process of acquisition. The complete HW setup used to capture microscopic data is shown in Fig. 1. All blood smear images within the dataset were captured under a magnification of 100× with an oil immersion objective lens and with an effective magnification of 1000. This resulted in an approximated resolution of 42 pixels per 1 μm. Since the distribution of leukocytes in the blood smear is usually uneven with the predominance of large leukocytes at distal parts of the blood smear and smaller cells in the center, systematic image acquisition was ensured by following the meander inspection pattern. This allowed us to capture microscopic images from different consecutive locations without omitting larger parts of the blood film. Most of the cells were captured from areas with evenly distributed cells and non-overlapping erythrocytes. However, to increase the variability of the dataset, cells from thicker, yet still relevant areas, were also included. The raw images were stored in an uncompressed bmp format with a 24-bit color depth and a resolution of 5472 × 3648 pixels.

### Ground truth labeling

The dataset annotation was performed manually by a trained person and under the supervision of a domain expert during the acquisition process. To accelerate this time-consuming process, we developed a customizable annotation tool with configurable labels and fully supported hotkey system allowing fast manipulation and operation with images. Within the labeling process, single cell images were cropped from each raw image, which reduced the final size of the images from 5472 × 3648 pixels to 1200 × 1200 pixels. Since using high-resolution technology comes with capturing a considerable amount of meaningless data, this approach of labeling not only optimized the memory consumption, but also reduced potential future computing load. The final dimension of the single cell images was derived on the basis of the biggest cells size within the collected samples. Besides reducing the image size, no filter and further preprocessing were performed to the images.

## Data Records

The dataset is publicly available at the Figshare data repository^17^. The dataset is organized into nine root folders, each representing one of the nine blood cell type, i. e., neutrophil segments, neutrophil bands, eosinophiles, basophiles, lymphocytes, monocytes, normoblasts (nucleated red blood cells), myeloblasts and lymphoblasts. Each root folder contains single-cell image data of a particular cell subtype, where each image has an uncompressed .bmp file format and is labeled with an incremental number.

The created white blood cell dataset contains a total of 10426 normal cells. To increase the variability of normal cells within the dataset, we included in the normal cell categories also mature cells from pathological and non-leukemic diagnoses. Besides the five basic normal cell categories, the dataset also contains a significant amount of diagnostically relevant immature cells of lymphoid and myeloid lineage. The total number of recorded immature blast cells amounts to 5091. The last part of the published dataset contains some of the less frequent cell classes, which presence in peripheral blood may be rare or might be unnoticed during the microscopic examination. These cell categories are neutrophil bands that precede neutrophil segments and rarely present immature red blood cells called nucleated red blood cells, which can be observed in peripheral blood smears during pathological conditions.

**Fig. 2:**
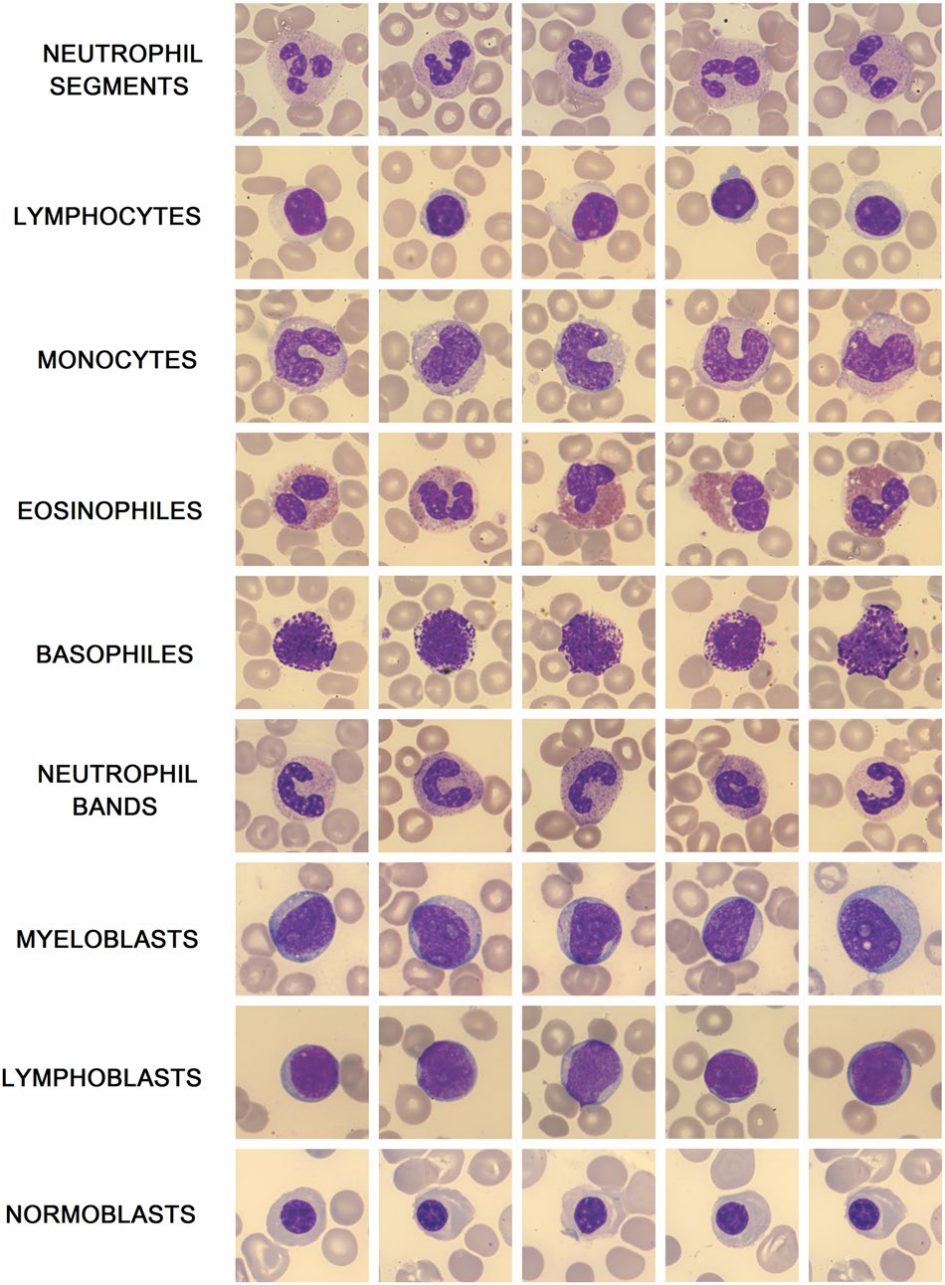
Typical samples of particular cell types within the dataset.

Although the clinical relevance of microscopic differences between myeloblasts and lymphoblasts is nowadays poor from a diagnostic point of view, as the cell differences can be subjective even for clinical specialists in some cases, we retained the types of blast cells in this dataset. This will allow further research in this specific area, where machine learning techniques have the potential to outperform human vision capabilities. Figure 2 shows dataset examples of all included cell categories.

## Technical Validation

**Fig. 3:**
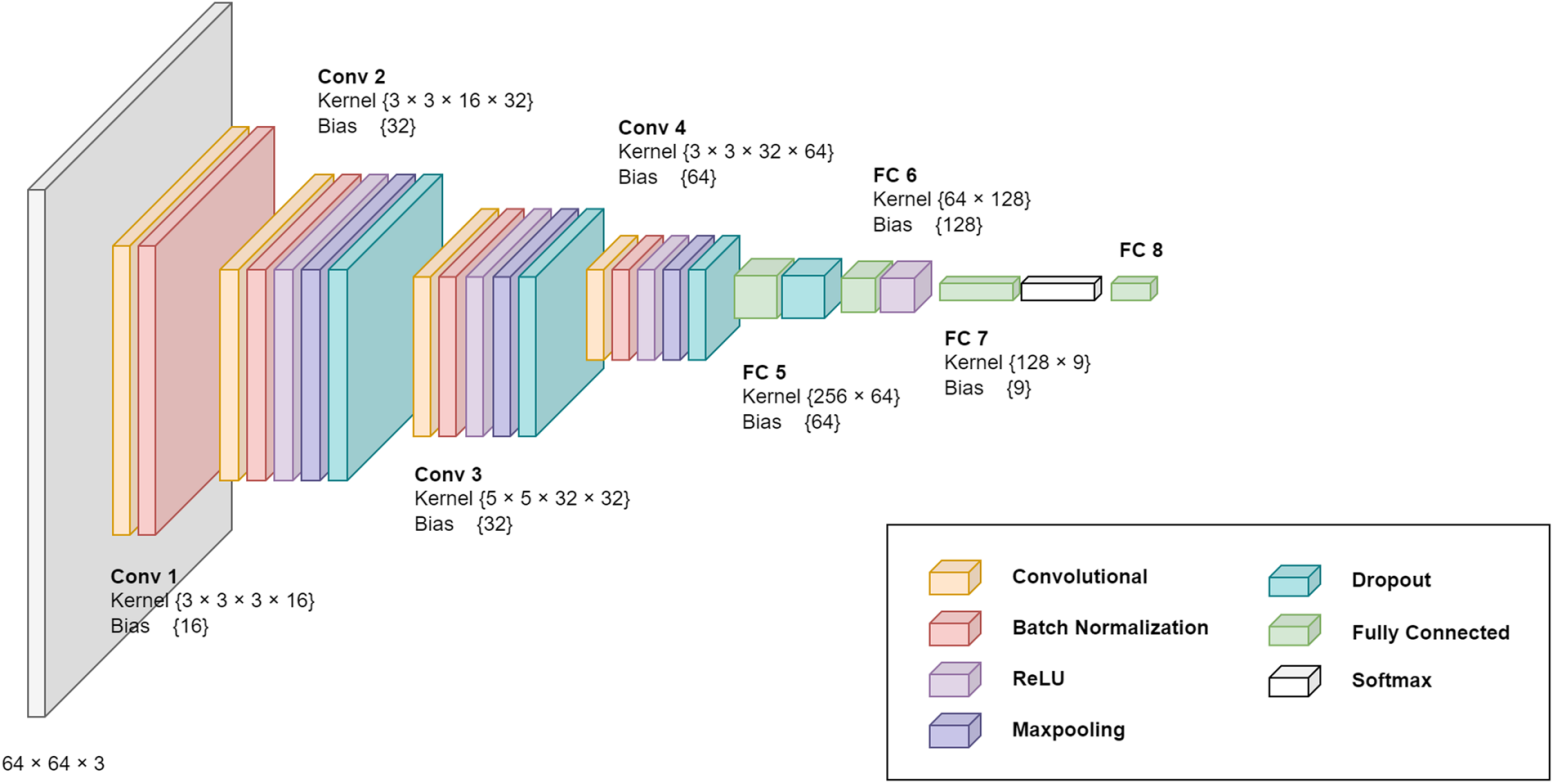
The proposed 8th layered CNN architecture.

To perform the technical validation of the dataset and evaluate the quality of the assigned labels, we developed and utilized a custom-made Convolutional Neural Network (CNN) detector. The proposed CNN architecture consists of a sequence of convolution layers alternated with max pooling, dropout, and batch normalization layers. The complete proposed architecture used in this study is depicted in Fig. 3.

The input layer of the network and all samples in the dataset were downsampled to a resolution of 64 × 64 × 3, where the last dimension represents the RGB layer of the input image. For training the neural network, we utilized the Adagrad optimization algorithm, which adjusts the learning rate of each parameter based on its past gradients, enabling the network to converge faster and enhance its accuracy over time. Additionally, we also applied data augmentation techniques to each sample in the dataset, including horizontal and vertical flip, translation (+-10 pixels in both directions), and scaling (+-0,02%). All the augmentation operations were applied with a 5% probability. The hold-out validation was then performed on a dataset, which was randomly divided into a training and testing set in a conventional ratio of 40/60.

At the beginning of the training, the neural network was initialized with the Xavier uniform initialization method for efficient and stable training. The model was then trained over a span of 85 epochs with a learning rate starting at 10^−3^ and gradually decreasing to 10^−4^ towards the end of the training, which facilitated an optimal convergence. The trained model resulted in an acceptable overall accuracy of 94.49%. Since the neural network outputted a significant number of misclassifications, all 530 misclassified samples were manually reviewed by a domain expert to verify that the misclassifications weren’t a result of incorrect annotations. The findings revealed that all misclassified samples were indeed a consequence of a failure of a classifier, which was primarily caused by the presence of blood film artifacts and their uneven distribution between the training and testing set. Because artifacts are an inevitable part of microscopic blood smear examination, further research must be conducted in the future to improve the accuracy of the classification.

## Data Availability

The customizable annotation tool used for the dataset labelling is available from https://github.com/AlexandraBodzas/Supporting-Material-for-Blood-Cells-Dataset.git.
